# Response to: increased prevalence of normal pressure hydrocephalus in both variants of frontotemporal dementia: a 10-year retrospective study

**DOI:** 10.1093/braincomms/fcad241

**Published:** 2023-09-15

**Authors:** Adrien de Guilhem de Lataillade, Philippe Damier, Hélène Pouclet-Courtemanche

**Affiliations:** INSERM, University Hospital of Nantes, CIC 1413, 44093 Nantes Cedex 1, France; Department of Neurology, University Hospital of Nantes, 44093 Nantes Cedex 1, France; Faculty of Medecine, Nantes Universite, 44093 Nantes Cedex 1, France; INSERM, University Hospital of Nantes, CIC 1413, 44093 Nantes Cedex 1, France; Department of Neurology, University Hospital of Nantes, 44093 Nantes Cedex 1, France; Faculty of Medecine, Nantes Universite, 44093 Nantes Cedex 1, France; INSERM, University Hospital of Nantes, CIC 1413, 44093 Nantes Cedex 1, France; Department of Neurology, University Hospital of Nantes, 44093 Nantes Cedex 1, France

We are very grateful to Dr. Pizzarotti and Prof. Allali^1^ for their interest in our article entitled: ‘Idiopathic normal pressure hydrocephalus and frontotemporal dementia: an unexpected association’^2^ and for this first replication of our study in a new cohort of frontotemporal lobar degeneration (FTLD) patients.

The main finding of their study is the high prevalence of idiopathic normal pressure hydrocephalus (iNPH) among a group of 45 FTLD (5 patients, 11.1%) which is even higher than in our population.

Among their five iNPH-FTLD patients, one presents a primary progressive aphasia (PPA), and four behavioural variant of FTLD (bv-FTLD). To our knowledge, this is the first description of an association of PPA with iNPH. It would have been interesting to know the detailed phenotype of this PPA patient. Indeed, PPA is a syndrome associated with several neuropathology: two of them, nonfluent/agrammatic and semantic variants, are mostly linked to FTLD (i.e. Tau-FTLD or TDP43-FTLD), and one of them, the logopenic variant, to Alzheimer’s disease.^3^

Since Prof Allali and collaborators have previously published a study focusing on Alzheimer’s disease and tap test in iNPH,^4^ we suppose that they should provide soon a measure of the prevalence of iNPH among their Alzheimer’s disease series. We look forward to comparing this prevalence with the prevalence that they reported in FTLD patients.

To conclude, this replication of our results in a 10-year study evaluating the prevalence of iNPH among 45 FTLD patients followed in another Memory Center reinforces the probable pathophysiological link between iNPH and FTLD. Moreover, neuropathological assessment of iNPH-FTLD patients should provide interesting cues to elucidate the relationship between neurodegenerative processes and iNPH.

## Data Availability

Data sharing is not applicable to this article as no new data were created or analysed.
